# Management of Posterior Shoulder Instability Among Football Players

**DOI:** 10.1007/s12178-025-09976-5

**Published:** 2025-05-14

**Authors:** Kira L. Smith, Luc Fortier, Andrew Moyal, John M. Apostolakos, Jacob G. Calcei, James E. Voos

**Affiliations:** Department of Orthopaedic Surgery, Drusinsky Sports Medicine Institute, University Hospitals Cleveland, 11100 Euclid Ave, Cleveland, OH 44106 USA

**Keywords:** Posterior shoulder instability, Return to play, Rehabilitation, Posterior capsulolabral repair

## Abstract

**Purpose of Review:**

Football players are at an increased risk for posterior shoulder instability compared to other sports due to certain sport-specific motions that involve posteriorly directed force on the shoulder in a vulnerable position. Management of posterior instability, both nonoperative and operative, is aimed at preventing recurrent instability. Regardless of treatment, timing of return to play revolves around avoiding reinjury and prioritizing player safety. This article provides a review of the current treatment modalities of posterior shoulder instability and the return to play criteria that must be met prior to releasing the player to competition.

**Recent Findings:**

Posterior shoulder instability was traditionally thought to occur predominately in offensive linemen. However, recent literature suggests it can also commonly be found in other players, including defensive linemen and quarterbacks. Current research reports high return to play rates for athletes that undergo arthroscopic posterior capsulolabral repair. Whereas, there is limited literature regarding return to play rates after posterior bony augmentation and management of reverse Hill-Sachs lesions. Rehabilitation and return to play protocols have been recommended but are nonspecific to American football.

**Summary:**

Posterior shoulder instability has the potential to sideline young athletes for an extended time and presents a complex challenge to both the athlete and the treating physician. Although the ultimate goal is to facilitate return to play, the physician must balance this with minimizing the risk of re-injury.

## Introduction

Posterior shoulder instability is an emerging topic of research that is less understood than anterior shoulder instability. Management of this pathology remains controversial as there is a relative paucity of high-level evidence on the topic [1]. A recent study aimed to establish consensus on the diagnosis, non-operative and operative treatment, as well as rehabilitation/return to play of posterior shoulder instability [1, 2]. Statements regarding diagnosis, nonoperative treatment, and labrum repair for posterior shoulder instability achieved strong or unanimous consensus 63% of the time [1, 2]. Statements related to glenoid-bone grafting, glenoid osteotomy, rehabilitation, return to play, and follow-up for posterior shoulder instability achieved strong or unanimous consensus 59% of the time [1, 2]. High quality literature on posterior shoulder instability is expanding but still remains relatively sparse, especially in the setting of specific athletic populations, including American football players.

### Epidemiology

Posterior shoulder instability makes up approximately 2 to 10% of all glenohumeral instability cases [3]. Within the general adult population the incidence of anterior shoulder dislocations has been reported to be between 17 and 23.9 per 100,000 person-years whereas the incidence of posterior shoulder dislocation is much less, estimated at 1.1 per 100,000 person-years [4–7]. Football players are at an increased risk to have posterior instability [8–10]. Among NCAA Division 1 football players who required shoulder surgery, 19% were for posterior instability [11]. Retrospective review of NFL Combine participants from 2009 to 2015 identified athletes with available shoulder magnetic resonance imaging (MRI) scans. Among players with shoulder MRIs, 34.7% to 38% showed evidence of posterior labral tears on MRI [12, 13]. In a study of NFL players that missed time due to shoulder instability injuries, 15% to 20% of those injuries were from a posterior instability event. [14] The median days missed due to a posterior instability event was 13 days (interquartile range 5–175 days) [14]. Alternatively, 54.3% of athletes had a posterior instability event but did not miss any time beyond the date of injury, highlighting the wide range of pathology of posterior shoulder instaiblity [15]. The majority of instability injuries occur during games via contact mechanism [10, 14–16].

The high rate of posterior instability among collegiate and professional football players is hypothesized to be the result of certain sport-specific motions that involve posterior directed force on a shoulder in a vulnerable position. Although previous research has identified that posterior instability more commonly affects linemen [12, 17–19], other positions with relatively high rates of posterior instability include defensive secondary players, quarterbacks, running backs, and tight ends [14–16].

### Mechanism

Posterior instability encompasses a broad spectrum of pathologies that affect the capsulolabral soft tissues and bony architecture of the shoulder. Rather than gross instability, such as subluxation or dislocation that is often described in anterior instability, vague pain without a definable injury is more characteristic of posterior instability [20]. The three mechanisms that have been proposed to cause posterior instability are 1) repetitive microtrauma, 2) acute traumatic posterior force, and 3) insidious onset laxity [21, 22]. Both repetitive microtrauma and laxity progress to chronic attenuation injuries of the posterior capsule and labrum while acute trauma can cause capsulolabral detachment. Repetitive posterior loading of a flexed, adducted, and internally rotated shoulder, as is seen with pass-blocking techniques, has been cited as the primary cause of posterior instability among football players [23–26]. Among quarterbacks, the late cocking and follow-through phases of the throwing motion induces repetitive stress on the posterior capsulolabral complex [27, 28]. The humeral head can translate posteriorly relative to the glenoid when the posterior capsulolabral complex has been compromised or is deficient. Glenoid retroversion, glenoid dysplasia, posterior humeral head subluxation, and increased capsular volume are also risk factors for the development of posterior instability in these athletes [19, 23, 29].

Furthermore, injuries to the posterior labrum are termed “reverse Bankart lesions”, while injuries to the posterior labrum with concomitant posterior glenoid rim fractures are termed “reverse bony Bankart lesions” [21]. Posterior shoulder dislocation events can also cause “reverse Hill-Sachs lesion”, which is used to describe an impaction injury to the anterior humeral head [30]. All of the previously described lesions are found to lead to an increased rate of recurrent instability [16, 21].

## Conservative Management

Non-operative management of posterior shoulder instability is typically the first line of treatment [24–26]. Surgery is reserved for those who do not respond to physical therapy or other conservative treatments. However, in high-risk, high-demand athletes, non-operative management becomes more complex. Recent research groups have suggested that being a collision athlete is a relative contraindication for pursuing non-operative management in treating posterior shoulder instability [1].

Nonoperative management is aimed at controlling pain and increasing stability. Cruz et al. recommends a 3-phase program, including 1) static proprioceptive control though closed-chain kinetic movements with visual feedback, 2) dynamization through isokinetic balancing of the internal and external shoulder rotators for global concentric strengthening, and 3) dynamic, proprioceptive, open-chain, kinetic exercises for eventual return to sports [31]. There are no comparative studies assessing various protocols to treat posterior shoulder instability.

There is a paucity of outcomes literature on football players with posterior shoulder instability who are treated conservatively. In a case series of 10 NCAA football players who initially opted for nonoperative management of their posterior shoulder instability, 7 out of 10 players returned to play in the same season, but later required surgery due to recurrent instability [32]. In a young athletic cohort of 113 patients, 77% of patients with posterior shoulder instability failed non-operative management and required surgical stabilization [9]. Murphy et al. found that athletes in the NFL combine who underwent surgical intervention for their posterior labral tear played a significantly higher percentage of snaps by their second NFL season compared to players treated nonoperatively [13]. Earlier surgical management may reduce recurrent instability and development of associated conditions such as complex labral tears and bone loss [33].

### Injury Prevention

To our knowledge, only one previous study has evaluated the role of functional shoulder bracing in preventing posterior instability [34]. Baker et al. evaluated 45 collegiate offensive linemen over multiple seasons who prophylactically utilized bilateral shoulder stabilizing braces (Donjoy Shoulder Stabilizer: Shoulder Pad Attachment). By limiting abduction, external rotation, internal rotation, and forced posterior subluxation the brace acted as an anchor to help prevent posterior glenohumeral instability. Braced athletes experienced a significantly decreased time lost due to injury, missing an average of 8.7 practices and/or games compared with 36.6 for non-braced athletes [34]. Although the authors found that braced athletes also experienced a decreased injury rate of posterior labral tears compared with non-braced athletes, these results were not statistically significant due to small sample size [34]. Furthermore, athletes have reported the psychological benefits of bracing include improved sense of stability and the ability to play at previous competition levels after injury [35–37]. These findings are further supported by biomechanical studies on proprioception [35–37].

## Operative Management

### Arthroscopic Posterior Capsulolabral Repair

Arthroscopic posterior capsulolabral repair is the most common used surgical treatment option for posterior shoulder instability. Previous literature reports high return to activity rates, low risk of recurrence, and clinically relevant improvements in patient reported outcomes (PROs) [3, 38]. Capsulolabral repair can be performed either with or without suture anchors. When the posterior labrum is partially or completely detached, suture-anchors are typically used to anchor the repaired soft tissue to the bony glenoid rim; whereas, cases of isolated significant posterior capsular laxity may be treated with sutures alone [28].

Arner et al. argues that arthroscopic capsulolabral repair for posterior shoulder instability is effective in football players and optimizes successful return to play due to improved stability, pain, and joint function [19]. The authors found that in a group of 56 consecutive American football players treated surgically with arthroscopic repair, 93% returned to sport and 79% returned to sport at the same level [19]. The athletes also had significant improvements between preoperative and postoperative pain, strength, function, stability, range of motion, and American Shoulder and Elbow Surgeon (ASES) scores. Although there was a 7.5% failure rate (defined as either ASES < 60 and stability score < 5), 96% of athletes were satisfied with their operation [19].

Of the 56 shoulders in Arner et al., 21% underwent capsulolabral plication without suture anchors, 55% had capsulolabral plication with suture anchors, and 23% had capsulolabral plication with suture anchors and additional plication sutures. Therefore, 79% of patients were treated with anchored fixation. The two cases of failures had a suture anchor repair construct while there were no failures in any of the patients treated with capsulolabral plication without suture anchors. Whether treated with anchored fixation or capsulorrhaphy alone, patients from both groups had similar improvements in ASES and stability scores [19]. Furthermore, rates of return to play were no different when comparing those who underwent anchored versus anchorless fixations, although this was limited by statistical power analysis [19]. The results indicate that constructs used during arthroscopic capsulolabral reconstructions should be customized to the pathoanatomy and individual athlete.

A study of 153 athletes in Division I football who underwent surgery for either anterior, posterior, or combined shoulder instability found no significant difference in return to play rates between the instability groups [39]. Of those who underwent posterior labral repair, 92.9% returned to play [39]. This rate is similar to previous research by Arner et al. and Bradley et al. who reported 93% and 89%, respectively [19, 27]. Bradley et al. has also reported low surgical revision rates for football players in several studies [27, 28, 40]. Reasons for revision included recurrent instability, continued pain, or decreased function. They found that decreased glenoid bone width was the only significant risk factor for requiring revision surgery [40, 41]. There was no significant difference in cartilage version, labral version, bone version, and labral width between patients that did and did not require revision surgery.

We are unaware of any studies evaluating the impact of time to surgery, those who undergo immediate surgery compared to those who undergo delayed surgery, and the risk of worsening injury or return to play rates.

### Additional Operative Management

Unlike anterior shoulder instability, the critical threshold of glenoid bone loss (GBL) and contribution of reverse Hill-Sachs lesions (RHSLs) in which bony augmentation should be considered during surgery has not yet been defined for posterior shoulder instability [42]. However, relative indications for posterior bony augmentation include notable GBL, high degrees of retroversion, insufficient posterior soft-tissue, or failure of arthroscopic capsulolabral repair [1, 2, 42]. Arner et al. found that in athletes with posterior instability that underwent arthroscopic repair, 11% GBL resulted in 10 times higher surgical failure rate and 15% GBL resulted in 25 times higher surgical failure rate [19]. It should be noted this study included athletes involved in any sporting activity at any level and was not specific to American football players. Interestingly, although increased glenoid retroversion has been cited as a risk factor for developing posterior instability, it is not necessarily a risk factor for surgical failure [16]. Postoperative outcome studies cite decreased bony width, not glenoid retroversion, as a factor leading to poorer surgical outcomes [41, 43]. However, it is important to note that this previous research is not specific to American football players.

To our knowledge, there is no current literature evaluating the outcomes of surgical treatment for RHSLs in the setting of posterior shoulder instability that is specific to an athletic population or American football players. Current recommendations include disimpaction versus bone grafting versus subscapularis tenodesis to address RHSLs with posterior instability [41]. A recent systematic review of 29 studies evaluating surgical treatment of RHSLs with posterior instability demonstrated low rates of recurrent instability as well as favorable clinical and patient-reported outcomes postoperatively [44]. However, the studies included in the systematic review were of patients in the general population not necessarily high level athletes. Most studies in this review reported on the modified open McLaughlin procedure, a technique that includes an osteotomy of the subscapularis insertion with fixation transferred into the RHSL defect, providing the advantage of bone-to-bone healing [45, 46].

## Rehabilitation and Return to Play Protocols

Although nonspecific to American football players, a recent study outlined the following criteria to consider for an athletes return to play after treatment of posterior shoulder instability; a) recovery of strength (> 90% of contralateral), b) recovery of range of motion (> 90% of contralateral), c) lack of apprehension, d) pain-free condition, e) sport-specific skills, and f) recovery of proprioception [2]. There was unanimous consensus that these criteria should be met following non-operative management with no minimum timeframe required before returning to play if the criteria are met [2]. There was strong consensus that following operative management these same criteria should be met and a minimum of 4 months is required before returning to play [2]. Unsurprisingly, the authors noted that collision athletes may take longer to return to play due to their high risk for recurrent instability, and more caution should be applied in allowing them to return to play. Elite athletes may have unique considerations in returning to play such as financial considerations, improved baseline health compared to the general population, and easier access to high-quality rehabilitation and medical evaluation [2].

To our knowledge, there is currently no comparative literature detailing different rehabilitation and return to play protocols specific to American football. However, examples of rehabilitation programs have been included [Tables 1, 2, 3].

**Table 1:** Example 1 of arthroscopic posterior capsulolabral repair rehabilitation protocol

Time	Exercises/Therapy
Weeks 0–3	‒ Sling in neutral rotation for 3 weeks (padded abduction sling) ‒ Codman exercises, elbow and wrist ROM ‒ Wrist and grip strengthening
Weeks 3–6	‒ Restrict to forward flexion 90°, internal rotation to stomach Progress from passive ROM to active assisted ROM to active ROM ‒ External rotation with arm at side as tolerated ‒ Begin isometrics with arm at side – FF/ER/IR/ABD/ADD ‒ Start scapular motion exercises (trapezius, rhomboids, levator scapulae) ‒ No cross-arm adduction, follow ROM restrictions ‒ Heat before treatment, ice after treatment per therapists’ discretion
Weeks 6–12	‒ Increase ROM to within 20° of opposite side; no manipulations per therapist; encourage patients to work on ROM daily ‒ Once 140° active FF, advance strengthening as tolerated: isometrics → bands → light weights (1–5 lbs); 8–12 reps/2–3 sets per rotator cuff, deltoid, and scapular stabilizers with low abduction angles ‒ Only do strengthening 3x/week to avoid rotator cuff tendonitis ‒ Closed chain exercises
Months 3–12	‒ Advance to full ROM as tolerated ‒ Begin eccentrically resisted motions, plyometrics (e.g. weighted ball toss), proprioception ‒ Begin sports related rehab at 3 months, including advanced conditioning ‒ Return to throwing at 4.5 months ‒ Push-ups at 4.5–6 months ‒ MMI is usually at 12 months post-op

**Table 2:** Example 2 of postoperative protocol following posterior stabilization

Time	General Guidelines	Goals	Exercises	Criteria to Progress
Phase 1: 0–2 wks	‒ Continuous rest in sling 24/7 in “Gunslinger” position ‒ Avoid elevation when supine, IR, horizontal adduction past neutral	‒ Protect posterior capsule ‒ Educate patient about surgical procedure and rehabilitation progression expectations ‒ Facilitate distal extremity exercises for circulation ‒ Minimize pain and inflammation	‒ Active elbow, wrist and hand ‒ Scapular retraction ‒ Cervical ROM, especially upper trapezius/levator scapula stretches	‒ Wound healing without infection ‒ Low to no pain
Phase 2: 2–6 wks	‒ Sling wear 24/7 until 4 weeks, then slow wean over 2 weeks, continuing to wear at night for 6 weeks ‒ Avoid FE > 90°, horizontal adduction past neutral, IR > 20°, ER > 30°	‒ Protect posterior capsule ‒ Initiate protected ROM to minimize stiffness ‒ Prevent muscle atrophy ‒ Resolve pain and inflammation	‒ PROM ER to 30° at 30°−45° abduction in scapular plane ‒ PROM IR to 20° at 30°−45° abduction in scapular plane ‒ Tabletop supported FE to 90° ‒ Submaximal isometrics for flexion, extension, abduction, ER in neutral ‒ NO IR isometric	‒ ER to 30° ‒ Elevation to 90° ‒ Clearance from surgeon to advance motion and wean from sling
Phase 3: 6–12 wks	‒ Avoid forced IR or horizontal adduction at end range ‒ Avoid closed chain exercises, or activities in WB position	‒ Restore full ROM (active and passive) in all planes, except ER ‒ Normalized biomechanics during elevation active ROM ‒ Increase rotator cuff and scapular stabilizer muscle strength	‒ Progressive passive ROM and stretching for elevation, ER(0) and ER(90) to end range ‒ Advance active assisted to active range for elevation (supine to incline to vertical; short to long lever arm; assisted to unassisted) ‒ Begin IR isometric exercise in slight ER – not past neutral ‒ TheraBand resisted ER, scapular stabilizers, deltoid, serratus without weightbearing ‒ Begin IR ROM to 45° in scapular plane	‒
Phase 4: 12–24 wks	‒ No interval throwing program or other overhead sport prior to 6 months ‒ No collision sport prior to 6 months ‒ No bench press or push-ups until 6 months	‒ Gradual restoration of IR and horizontal adduction ROM toward symmetry of uninvolved side ‒ Increase strength, power, endurance of entire shoulder girdle ‒ Improve proprioception and neuromuscular control ‒ Integrate trunk needs for motion and strength that are sport specific	‒ Add IR TheraBand to program with aim in neutral position ‒ Add gentle posterior capsule stretches such as cross body, hand slide up spine, sleeper stretch ‒ Continue ER, scapular stabilizer, deltoid, biceps, triceps slow progressive resistive exercise ‒ Add diagonal patterns and eccentric workouts as strength of cuff below shoulder level normalizes ‒ When strength is normalized, add plyometric ball tosses, body blade (4–5 months)	‒ Cleared by surgeon ‒ Full active ROM all planes of shoulder motion with normalized mechanics ‒ Normalized muscle strength proportional to work/sport demands

**Table 3:** Example 3 of arthroscopic posterior capsulolabral repair rehabilitation protocol [28]

Time	Exercises/Therapy
Immediately after surgery	‒ Place in a sling that immobilizes the shoulder in ~ 30° of abduction while preventing internal rotation
1–3 days	‒ Active wrist and elbow motion ‒ Gentle passive scaption exercises ‒ Crytotherapy used for edema control
7–10 ays	‒ First postoperative visit
4–6 weeks	‒ Sling immobilization discontinued ‒ Active elevation of the arm ‒ Gentle passive range of motion exercises advanced to active assisted range of motion ‒ Gentle, pain-free internal rotation and external rotation ‒ Isometric internal rotation and external rotation ‒ Sling immobilization discontinued depended on amount of capsular laxity found at time of surgery
2 to 3 months	‒ Full passive and active range of motion ‒ Capsular strengthening exercises ‒ Isotonic strengthening continued with emphasis on rotator cuff and posterior deltoid
6 months	‒ Isokinetic testing performed ‒ Once the patient is able to achieve 80% strength and endurance compared with the contralateral side, sport specific rehabilitation protocol initiated ‒ Athletes required to achieve full ROM without pain, full strength, and endurance comparable with the contralateral side*

^*^Throwing athletes deserve special consideration and accordingly were placed on a specific protocol in which their throwing distance and speed were closely monitored and slowly advanced over 2 to 3 months. Once the throwing athlete was able to perform full-speed throwing for 2 consecutive weeks without symptoms, return to full competition was permitted

## Conclusion

Posterior shoulder instability is an expanding area of research. Players most at risk for posterior shoulder instability and posterior labrum tears are those that endure posterior directed force on the shoulder while it’s in a vulnerable position. Although operative intervention is generally more favorable in this population, few games are missed during the season due to posterior shoulder instability when managed non-operatively. After surgical intervention, there is a high rate of return to play typically after 4 months of recovery and rehabilitation. Recurrent posterior shoulder instability is associated with increased GBL, although a definitive threshold for bone grafting is not yet known.Future research should consider treatments that can tolerate the demands of the sport, incorporate the athlete’s pathoanatomy and position specific demands, as well as develop more precise and distinct functional outcome methods.

## Key References

- Arner JW, McClincy MP, Bradley JP. Arthroscopic Stabilization of Posterior Shoulder Instability Is Successful in American Football Players. Arthroscopy. 2015;31(8):1466–1471. 10.1016/j.arthro.2015.02.022
  - ⚬ This paper provides valuable functional and clinical outcome data on the population of interest (football players) after posterior arthroscopic labral repair
- Baker HP, Tjong VK, Dunne KF, Lindley TR, Terry MA. Evaluation of Shoulder-Stabilizing Braces: Can We Prevent Shoulder Labrum Injury in Collegiate Offensive Linemen?. Orthop J Sports Med. 2016;4(12):2325967116673356. Published 2016 Dec 1. 10.1177/2325967116673356
  - ⚬ This paper is the only one to our knowledge that assesses conservative management of posterior shoulder instability in football players
- Bradley JP, Baker CL 3rd, Kline AJ, Armfield DR, Chhabra A. Arthroscopic capsulolabral reconstruction for posterior instability of the shoulder: a prospective study of 100 shoulders. Am J Sports Med. 2006;34(7):1061–1071. 10.1177/0363546505285585
  - ⚬ To our knowledge, this is one of the first papers to investigate arthroscopic capsulolabral reconstruction for posterior shoulder instability
- Hurley ET, Aman ZS, Doyle TR, et al. Posterior Shoulder Instability, Part I-Diagnosis, Nonoperative Management, and Labral Repair for Posterior Shoulder Instability-An International Expert Delphi Consensus Statement. *Arthroscopy*. Published online May 11, 2024. 10.1016/j.arthro.2024.04.035
  - ⚬ This is a recently published consensus statement from 71 shoulder/sports surgeons from 12 countries on the diagnosis and management of posterior shoulder instability. Although nonspecific to football players, there is currently no other literature dedicated to clearly defining diagnosis and management of posterior shoulder instability
- Hurley ET, Aman ZS, Doyle TR, et al. Posterior Shoulder Instability Part II—Glenoid Bone-Grafting, Glenoid Osteotomy, and Rehabilitation/Return to Play—An International Expert Delphi Consensus Statement. *Arthroscopy*. Published online May 10, 2024.
  - ⚬ This is a recently published consensus statement from 71 shoulder/sports surgeons from 12 countries on return to play after posterior shoulder instability. Although nonspecific to football players, there is currently no other literature dedicated to clearly defining the various surgical options and return to play criteria related to posterior shoulder instability
- Robins RJ, Daruwalla JH, Gamradt SC, et al. Return to Play After Shoulder Instability Surgery in National Collegiate Athletic Association Division I Intercollegiate Football Athletes. *Am J Sports Med*. 2017;45(10):2329–2335. 10.1177/0363546517705635
  - ⚬ This paper provides important information regarding return to play after posterior shoulder instability specific to our population of interest (football players)

## Data Availability

No datasets were generated or analysed during the current study.
